# A prospective follow-up study of the relationship between high-sensitivity C-reactive protein and primary liver cancer

**DOI:** 10.1186/s12885-020-07665-9

**Published:** 2020-11-30

**Authors:** Sarah Tan Siyin, Tong Liu, Wenqiang Li, Nan Yao, Guoshuai Xu, Jun Qu, Yajun Chen

**Affiliations:** 1 Department of General Surgery, Aerospace Center Hospital, Yuquan Road 13, Haidian District, Beijing, 100089 China; 2 Department of General Surgery, Beijing Children’s Hospital, National Center for Children’s Health, Beijing, China

**Keywords:** Primary liver cancer, Incidence, High-sensitivity C-reactive protein, Competing risk models, Cohort

## Abstract

**Background:**

Competing risk method has not been used in a large-scale prospective study to investigate whether increased levels of high-sensitivity C-reactive protein (hs-CRP) elevate the risk of primary liver cancer (PLC). Our study aims to prospectively investigate the relationship between hs-CRP and new-onset PLC.

**Methods and results:**

Ninety-five thousand seven hundred fifty-nine participants without the diagnosis of PLC, and who had their demographic characteristics and biochemical parameters recorded, were analyzed from the Kailuan Cohort study. Cox proportional hazards regression models and competing risk regression models were used to evaluate the hazard ratios (HRs) and 95% confidence intervals (95% CIs) of PLC. During a median follow-up of 11.07 years, 357 incidental PLC cases were identified over a total of 1,035,039 person-years. The multivariable HRs (95%CI) for the association of hs-CRP of 1–3 mg/L group and hs-CRP>3 mg/L with PLC were 1.07(0.82 ~ 1.38), 1.51(1.15 ~ 1.98) in a Cox proportional hazard regression analysis adjusted for other potential confounders. In the cause-specific hazard model, the multivariable HRs (95%CI) for the association of hs-CRP of 1–3 mg/L group and hs-CRP>3 mg/L with PLC were 1.06(0.81 ~ 1.40), 1.50(1.14 ~ 1.99). Similar results were also observed in the sub-distribution hazard function model with corresponding multivariate HRs (95%CI) of 1.05(0.80 ~ 1.40), 1.49(1.13 ~ 1.98) in hs-CRP of 1–3 mg/L group and hs-CRP>3 mg/L group, respectively.

**Conclusions:**

This prospective study found a significant association of higher levels of hs-CRP with new-onset PLC. The main clinical implications would be an increased awareness of hs-CRP and its correlation to the risk of PLC. This study should be a steppingstone to further research on chronic inflammation and PLC.

**Trial registration:**

**Registration number:** ChiCTR–TNRC–11001489.

## Background

Primary liver cancer (PLC) is well recognized as one of the leading causes of cancer-related death globally, with hepatocellular carcinoma (HCC) and intrahepatic cholangiocarcinoma (ICC) being the most common, accounting for approximately 70 and 15% respectively [1]. World Health Organization revealed 841,080 new incidents and 781,631 deaths of PLC in 2018 worldwide. PLC incidence rates vary geographically, with East and South-East Asia consistently having the highest rates and regions in Oceania having the lowest [2]. China boasts of 19% of the world’s population but comprises of over 50% of PLC incident and death rates [3]. These high rates are mainly due to chronic infection of hepatitis B virus (HBV), affecting approximately 7.2% of the Chinese population [4]. Other known risk factors for PLC include hepatitis C virus (HCV), consumption of aflatoxin-contaminated foods, history of liver disease, diabetes and alcohol consumption [5–8].

Subsequent epidemiologic studies have demonstrated that chronic inflammatory processes are closely associated with several types of cancers [9–12]. C-reactive protein is an acute-phase reactant and is a marker for systemic inflammation. High-sensitivity C-reactive protein (hs-CRP) can detect small amounts of serum CRP, even within the normal range, and differs from CRP only in analytical sensitives and assay range [13]. Recognized as an independent cardiovascular risk factor [14, 15], the increment of hs-CRP also plays an important role in the pathogenesis of several cancers, specifically lung, colorectal and gastric cancer [10–12]. It has also been hypothesized that CRP may have an etiologic role in the occurrence of PLC, but there are limited studies available and the relationship between hs-CRP and new-onset PLC is not well established [16–18]. However, a competing risk method has not been used by large-scale prospective study to ascertain if increased levels of hs-CRP elevate the risk of PLC. Our study involved over 11 years of data from the Kailuan Study (Trial identification: ChiCTR–TNRC–11001489; Registration number:11001489) and used different risk models that were adjusted for traditional risk factors to prospectively investigate the relationship between hs-CRP and new-onset PLC.

## Methods

### Kailuan study

The data was obtained from the Kailuan Study, which is a prospective cohort study based on the population of the Kailuan community in Tangshan (Hebei Province), 150 km southeast of Beijing. Kailuan Group, a coal industry company, has branched out into a range of other fields including healthcare, education, manufacture, etc. This study was designed to investigate the risk factors associated with chronic diseases such as cancers, arteriosclerosis, hypertension, and diabetes mellitus. From July 2006 to October 2007, all 155,418 employees (including retirees) aged 18 to 98 years from Kailuan Corporation were invited to participate the physical examinations (the baseline examination) at Kailuan General Hospital and its 10 affiliated hospitals. A total of 101,510 participants (65.3%) aged 18–98 years agreed and were enrolled after written informed consent was obtained. All participants were then followed up biennially to collect information of potential risk factors and newly diagnosed PLC cases [19].

### Participants

**Fig. 1:**
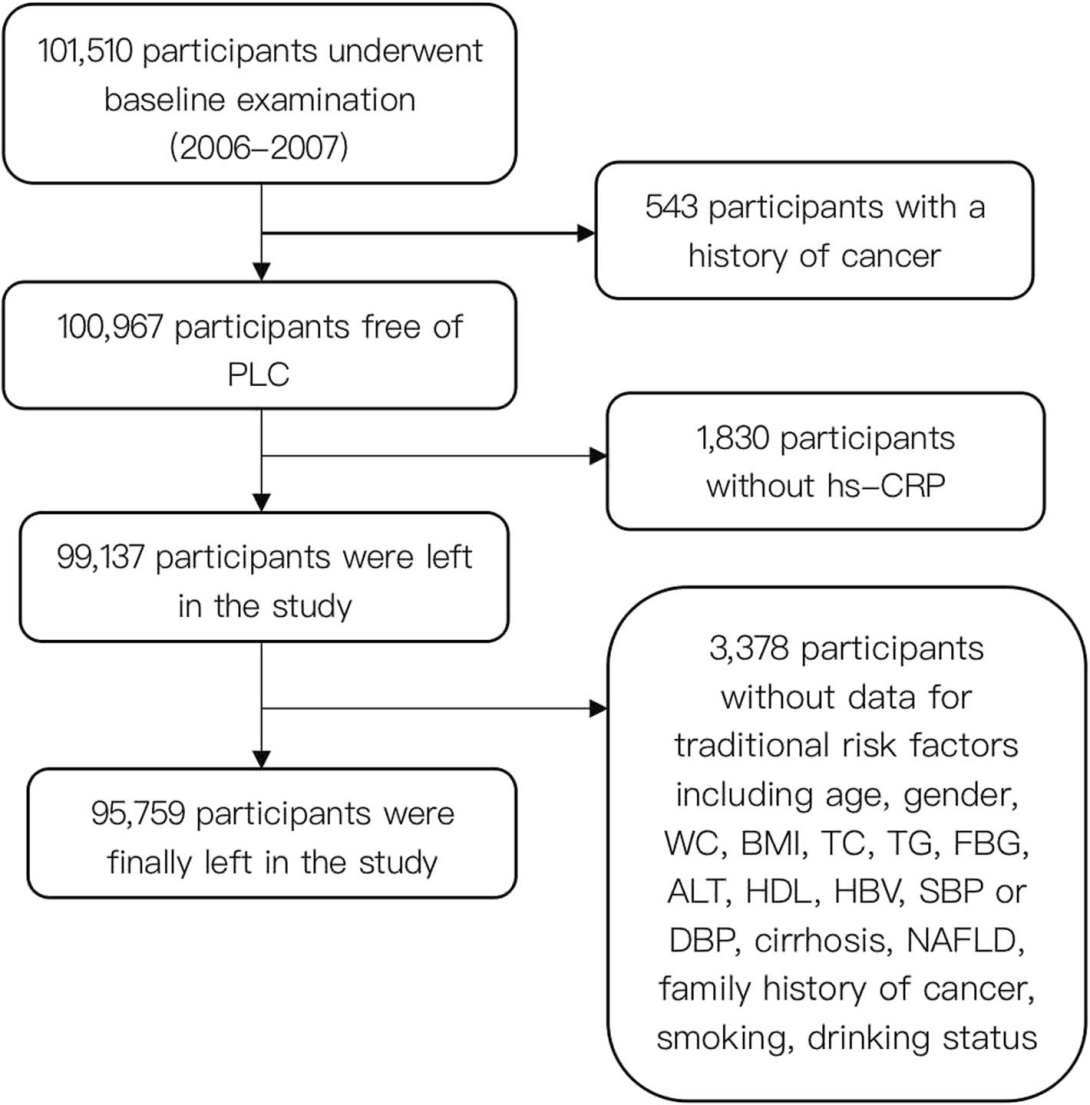
The procedure of participants screening

In this study, 543 participants with a diagnosis of PLC, 1830 participants without hs-CRP measurement, and 3378 participants without measurements for traditional risk factors for PLC at the baseline examination were excluded. Traditional risk factors include age (*N* = 735), gender (*N* = 443), waist circumference (WC, in cm, *N* = 258), body mass index (BMI, in Kg/m^2^, *N* = 312), total cholesterol (TC, in mmol/L, *N* = 133), triglyceride (TG, in mmol/L, *N* = 290), fasting blood glucose (FBG, in mmol/L, *N* = 90), alanine aminotransferase (ALT, in u/L, *N* = 107), high-density lipids (HDL, in mmol/L, *N* = 88), low-density lipids (LDL, in mmol/L, *N* = 52), HBV infection (*N* = 242), systolic blood pressure or diastolic blood pressure (SBP or DBP, *N* = 44), cirrhosis (*N* = 193), nonalcoholic fatty liver disease (NAFLD, *N* = 83), family history of cancer (*N* = 59), smoking (*N* = 113) and drinking status (*N* = 87) and physical activity (*N* = 49). The remainder of 95,759 participants (76,540 males and 19,219 females) participated in this study. Those who were excluded in this study were older (54.13 ± 11.30 years versus 53.12 ± 9.12 years, *P* < 0.001), and had higher levels of SBP (133.75 ± 19.15 mmHg versus 132.12 ± 20.12 mmHg, *P* < 0.001), BMI (26.19 ± 4.07 Kg/m^2^ versus 25.63 ± 3.23 Kg/m^2^, *P* < 0.001), WC (87.92 ± 11.38 cm versus 86.33 ± 11.40 cm, *P* < 0.001), and exhibited higher prevalence of cirrhosis (8(0.14) versus 97(0.10), *P* < 0.001) and HBV infection + (167(2.90) versus 2616(2.73), *P* < 0.001). The details of the participants’ screening were shown in Fig. 1. Based on guidelines from Disease Control and Prevention and the American Heart Association, participants were divided into three groups according to their hs-CRP concentration: < 1 mg/L, 1-3 mg/L, and > 3 mg/L [20]. This study complied with the Declaration of Helsinki and was approved by the Ethics Committee from both Kailuan General Hospital, Aerospace Center Hospital, and Beijing’s Children Hospital. Informed consent was obtained from all individual participants included in the study.

### Questionnaire assessment

Participants had in person interviews with qualified physicians and nurses to complete a standardized questionnaire to obtain their baseline information. Information obtained from the questionnaire at baseline included age, gender, family history of cancer, smoking status, drinking status, and physical activity level. Drinking was classified as having consumed at least 100 ml/day of alcohol for longer than 6 months. Smoking was classified as having at least 1 cigarette/day for longer than 6 months. Evaluation of physical activity was carried out using responses regarding the frequency of physical activity (≥3 times/week, ≥30 min/time).

### Anthropometric measurements and blood pressure measurement

During baseline and all subsequent biennial interviews, height, weight, WC and blood pressure (BP) measurements for the participants were taken. A tape rule was used to measure height and was rounded to the nearest 0.1 cm. Weight was rounded to the nearest 0.1 kg. A calculation of weight/square of height was carried out to obtain body mass index (BMI). WC was measured at the narrowest point between the lowest rib and pelvis during expiration and was rounded to the nearest 0.1 cm. Blood pressure was taken when participants were seated and with a 5 min interval in between. The average of the two readings, both taken from the left arm, was used for analysis. Hypertension was classified as having a history of hypertension, systolic blood pressure ≥ 140 mmHg, diastolic blood pressure ≥ 90 mmHg, or the use of antihypertensive medications.

### Laboratory assessment

Vacuum tubes with EDTA were used to obtain overnight (≥8 h) fasting venous blood samples. Separation of the plasma was carried out, and plasma was kept at − 80 °C for further analysis. Hs-CRP concentrations were measured using a commercial, high-sensitivity particle-enhanced immunonephelometry assay (Cias Latex CRP-H, Kanto Chemical Co. Inc., Tokyo, Japan). The lower limit of detection was 0.1 mg/L. TC and TG concentration was measured using the colorimetric enzymatic method (Mind Bioengineering Co Ltd., Shanghai, China). The upper limit of detection was 20.68 and 11.30 mmol/L, respectively. LDL-C and HDL-C concentration were measured using the direct test method (Mind Bioengineering Co. Ltd., Shanghai, China), and there was a respective detectable upper limit of 12.9 and 3.88 mmol/L. The hexokinase/glucose-6-phosphate dehydrogenase method and the enzymatic method was used to measure FBG and ALT respectively. The inter-assay coefficient of variation for each measurement was lower than 10%. All plasma samples were analyzed using an auto-analyzer (Hitachi 747; Hitachi, Tokyo. Japan) at the central laboratory in Kailuan General Hospital. Diabetes mellitus was defined as a fasting blood glucose level ≥ 7.0 mmol/L, taking oral hypoglycaemic agents or insulin, or a validated physician diagnosis.

### Outcome ascertainment

All PLC cases were identified via the subsequent biennial follow-up, either through clinical examinations or in person interviews, and were classified according to ICD-10 numbers. Additional information from Kailuan General Hospital and the 10 affliated hospitals’ medical records were taken into consideration to prevent missed diagnosis. In cases where outcome information of participants were unavailable, death certificates from the Provincial Vital Statistics Offices (PVSO) were also taken into consideration. Review of medical records and consultation of post-surgical pathology reports confirmed PLC diagnosis.

### Statistical analysis

A commercially available software program (SAS software, version 9.4) was used to complete the statistical analysis in this study. Calculation of person-year was based on the date of their first examination until the first occurrence of the following circumstances: date of PLC diagnosis, death, or end of follow up (31 December 2018). Mean ± standard deviation was used to describe normally distributed variables and one-way analysis of variance (ANOVA) was used to make comparisons among subgroups. Median (interquartile range) was used to describe the skewed distribution (TG and ALT) and compared using nonparametric tests. Categorical variables were represented by percentage and the χ^2^ test was used for comparison among groups. Kaplan-miere was used to calculate the cumulative incidence of PLC by hs-CRP, and the differences of cumulative incidence were tested by log-rank test [19]. The associated risk of hs-CRP for new-onset PLC was estimated using Cox proportional hazard models, and hazard ratios (HRs) and 95% confidence intervals (95% CI) were calculated. Adjustments for confounding variables were made when fitting three models. Model 1 was a univariate analysis, model 2 was adjusted for age and sex, and model 3 was further adjusted for BMI, ALT, cirrhosis (yes/no), HBV infection (positive/negative), NASH/NAFLD (yes/no), alcoholic liver disease (yes/no), diabetes mellitus (yes/no), family history of cancer (yes/no), smoking status (yes/no), drinking status (yes/no), and physical activity (yes/no). We performed tests for linear trends by entering the median value of each hs-CRP category in the models. Acknowledging that epidemiologic data is limited because competing events (death) can preclude PLC from occurring, in the presence of competing risks, the traditional multivariate COX regression model has the potential to overestimate the absolute risk. For extrapolation purposes, prognostic models that estimate individual risk need to be as precise as possible. Because all-cause death (except PLC related death) may preclude the occurrence of PLC, the existence of competing events might lead to inaccurate estimation of the risk in traditional multivariate COX regression. A more realistic method of estimating the relationship between hs-CRP and new-onset PLC is the application of competing risk models. Application of the cause-specific hazard model (CS model) is suitable when studying the etiology of disease, whereas the sub-distribution hazard function model (SD model) are more suitable when predicting the outcome risk of an individual, indicating the fundamental difference between these two models [21, 22]. Thus, competing risk regression models (CS and SD model) were used to calculate the absolute risk of PLC. To reconfirm the association of hs-CRP with the risk of PLC, subgroup analyses were carried out by stratifying participants according to sex, age (in years) (youth: ≤45, middle age: 45–59 and old age: ≥60) or several important risk factors, including HBV infection (positive/negative) and cirrhosis (yes/no). The dose-response association of hs-CRP with PLC was calculated by restricted cubic spline regression (RCS). Statistical tests were 2-sided, and *P* less than 0.05 was deemed as statistically significant.

## Results

### Population characteristics

The mean age at baseline was 51.69 ± 12.48 years for the study population, of which 76,540 (79.9%) were males. Participants in the hs-CRP < 1 mg/L group had a greater prevalence of HBV (+) and higher alcohol and tobacco consumption. Participants in the hs-CRP of 1-3 mg/L group had the highest DBP, BMI, TC, TG, ALT, LDL-C levels, the highest prevalence of a family history of cancer and physical activity. Participants in hs-CRP > 3 mg/L group were older, with the highest SBP, WC, FBG, and HDL-C and with a higher prevalence of hypertension, diabetes, cirrhosis, and NAFLD. Baseline characteristics of participants stratified by hs-CRP levels (< 1 mg/L, 1-3 mg/L, and > 3 mg/L) are showed in Table 1.

**Table 1:** Baseline characteristics of the participants stratified by hs-CRP subgroups

	hs-CRP				
	< 1 mg/L	1–3 mg/L	> 3 mg/L	F/X^**2**^	***P-*** value
**Number**	**52,867**	**24,522**	**18,370**		
**Age (Year)**	**49.99 ± 12.08**	**52.40 ± 12.58**	**55.63 ± 12.48**	**1494.91**	**< 0.0001**
**Male (N)**	**42,603 (80.59)**	**19,602 (79.94)**	**14,335 (78.03)**	**55.28**	**< 0.0001**
**Sbp (mmHg)**	**129.08 ± 20.23**	**133.25 ± 21.54**	**134.27 ± 22.08**	**585.20**	**< 0.0001**
**Dbp (mmHg)**	**82.89 ± 11.55**	**84.54 ± 11.92**	**84.47 ± 12.10**	**225.80**	**< 0.0001**
**WC (cm)**	**85.29 ± 9.55**	**88.52 ± 9.73**	**90.14 ± 10.50**	**2046.10**	**< 0.0001**
**BMI (Kg/m**^**2**^**)**	**24.54 ± 3.31**	**25.76 ± 3.48**	**25.64 ± 3.76**	**1370.75**	**< 0.0001**
**TC (mmol/L)**	**4.90 ± 1.15**	**5.04 ± 1.15**	**4.96 ± 1.13**	**115.10**	**< 0.0001**
**TG (mmol/L)**	**1.21 (0.85 ~ 1.81)**	**1.38 (0.98 ~ 2.08)**	**1.35 (0.94 ~ 2.06)**	**1008.99**	**< 0.0001**
**FBG (mmol/L)**	**5.38 ± 1.49**	**5.60 ± 1.80**	**5.62 ± 1.99**	**203.33**	**< 0.0001**
**ALT (u/L)**	**18.12 (13.65 ~ 24.08)**	**19.63 (13.00 ~ 26.13)**	**18.09 (12.90 ~ 25.12)**	**252.39**	**< 0.0001**
**HDL-C (mmol/L)**	**1.56 ± 0.39**	**1.52 ± 0.39**	**1.58 ± 0.45**	**63.64**	**< 0.0001**
**LDL-C (mmol/L)**	**2.39 ± 0.78**	**2.45 ± 0.84**	**2.10 ± 1.26**	**877.22**	**< 0.0001**
**HBV infection + (N)**	**1519 (2.91)**	**671 (2.77)**	**426 (2.34)**	**16.43**	**0.0003**
**Hypertension (N)**	**20,741 (39.23)**	**11,850 (48.32)**	**9459 (51.49)**	**1092.35**	**< 0.0001**
**Diabetes (N)**	**3736 (7.07)**	**2710 (11.05)**	**2253 (12.26)**	**600.39**	**< 0.0001**
**Cirrhosis (N)**	**38 (0.07)**	**32 (0.13)**	**27 (0.15)**	**10.38**	**0.0056**
**NAFLD (N)**	**13,533 (25.67)**	**9559 (39.14)**	**7405 (40.42)**	**2139.65**	**< 0.0001**
**Family history of cancer (N)**	**1756 (3.32)**	**1045 (4.26)**	**624 (3.40)**	**45.05**	**< 0.0001**
**Smoking status (N)**	**16,613 (31.42)**	**7891 (32.18)**	**4617 (25.13)**	**303.65**	**< 0.0001**
**Drinking status (N)**	**9840 (18.61)**	**4519 (18.43)**	**2562 (13.95)**	**217.05**	**< 0.0001**
**Physical activity (N)**	**7746 (14.65)**	**4529 (18.47)**	**2471 (13.45)**	**253.56**	**< 0.0001**

*SBP* Systolic blood pressure, *DBP* Diastolic blood pressure, *WC* Waist circumference, *BMI* Body Mass Index, *TC* Total cholesterol, *TG* Triglycerides, *FBG* Fasting blood glucose, *ALT* Alanine aminotransferase, *HDL-C* High-density lipoprotein cholesterol, *LDL-C* Low-density lipoprotein cholesterol, *HBV+* Hepatitis B virus infection, *NAFLD* Non-alcoholic fatty liver disease

TG and ALT were skewed distributed variables and presented as median (interquartile range)

### Incidence of PLC

**Fig. 2:**
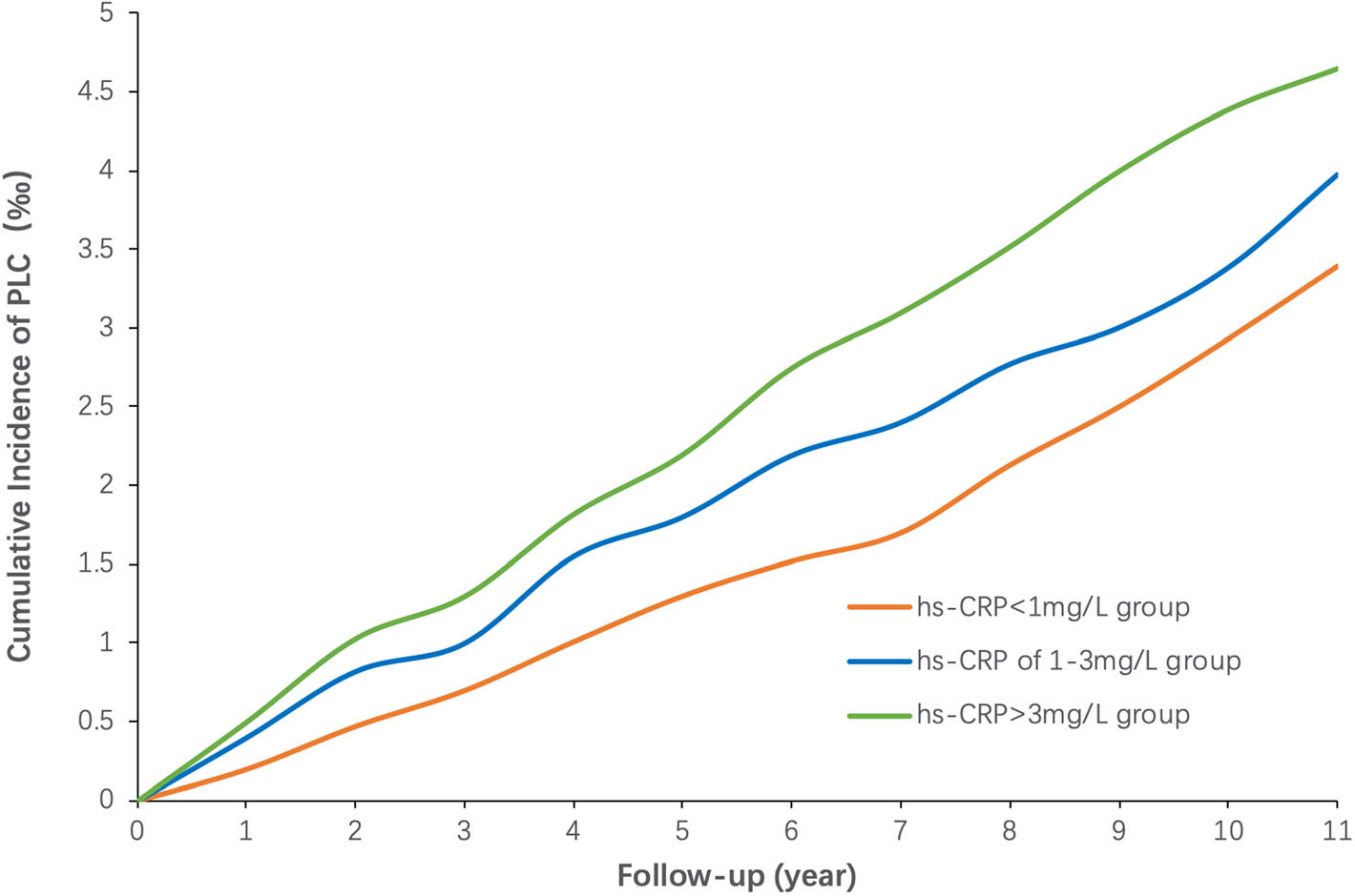
Cumulative incidence of PLC stratified by hs-CRP

The median follow-up time was 11.07 years per participant. At the end of the study, 357 new-onset PLC cases were diagnosed among 95,759 participants. The crude incidence per 10,000 person-years of PLC was 3.45 in all participants (4.03 in male participants, 1.11 female participants). Age- and sex- standardized incidence of PLC increased from 2.73 to 3.14 and 4.07 per 10,000 person-years in the hs-CRP < 1 mg/L, hs-CRP of 1-3 mg/L and CRP > 3 mg/L group, respectively. Figure 2 is a smooth model-based graph and describes the details of the cumulative incidence of PLC stratified by hs-CRP. The log-rank test indicated that the cumulative incidence of PLC among different hs-CRP subgroups had a significant statistical difference where the incidence increased as hs-CRP levels increased.

### Association of hs-CRP with the risk of PLC

**Fig. 3:**
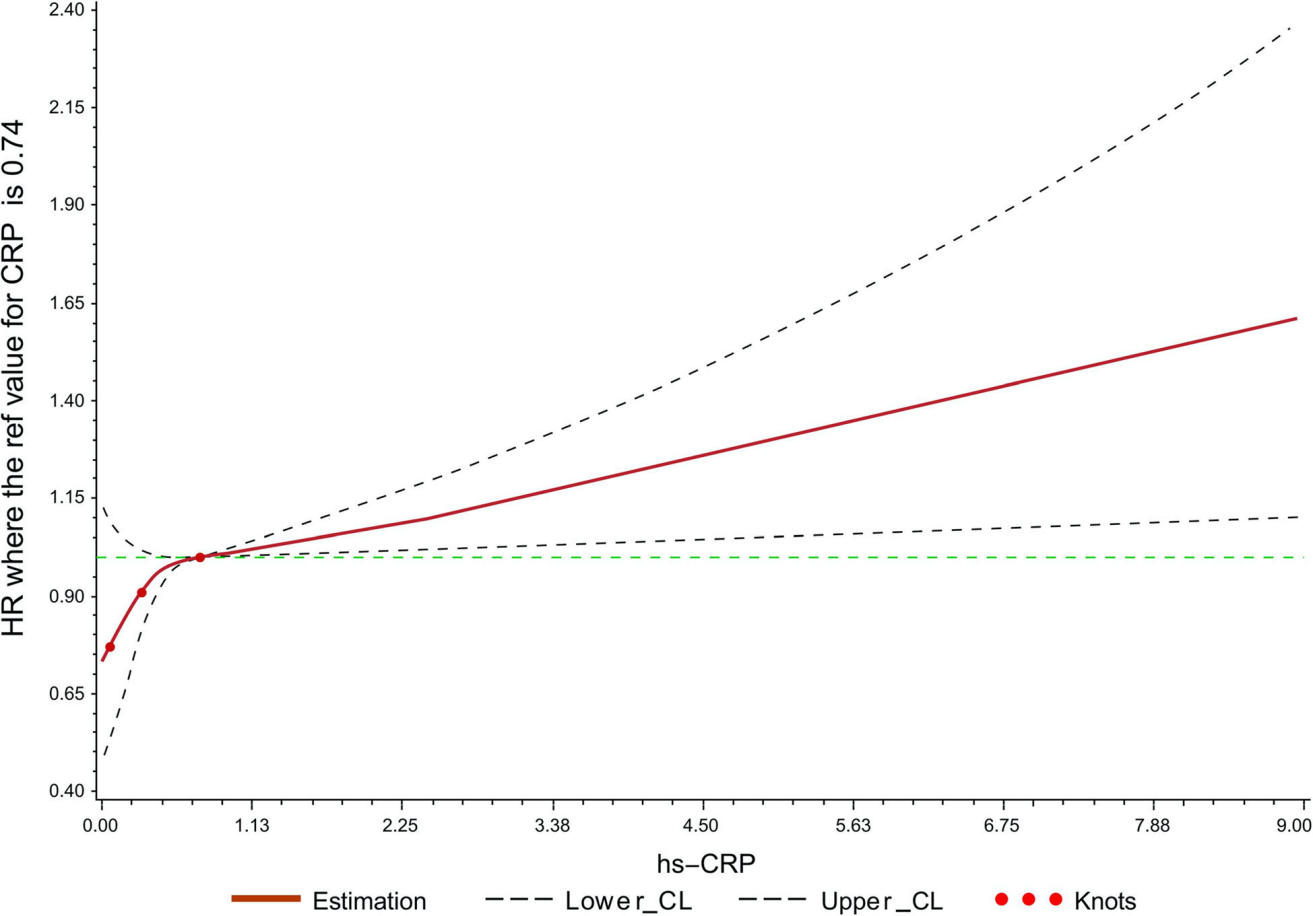
Association between PLC and hs-CRP using RCS with 3 knots. Cubic spline graph of the adjusted HR (represented by solid line) and 95%CI (represented by the dotted lines)

Commonly used categories for hs-CRP were analyzed in univariate and multivariate proportional hazards analyses to test for associations of risk for new PLC events (Table 2). Compared with CRP < 1 mg/L group, no significant associations were observed for hs-CRP of 1–3 mg/L on PLC risk in the univariate analysis (HR, 1.08; 95%CI, 0.84 to 1.40) and multivariate analysis (HR, 1.07; 95%CI, 0.82 to 1.38). Statistically significant increased crude and multivariable HRs were observed in people with CRP > 3.0 mg/L for PLC with corresponding HRs (95% CI) of 1.38(1.06 ~ 1.81) and 1.51(1.15 ~ 1.98), respectively. A positive linear association between hs-CRP levels and the risk of PLC was observed in the RCS model showed (P-overall = 0.0037, P-nonlinear = 0.0550; Fig. 3).

**Table 2:** Hazard ratios and 95% confidence interval (CI) for risk of PLC among participants stratified by hs-CRP subgroups in different regression models

	hs-CRP			
	< 1 mg/L	1–3 mg/L	> 3 mg/L	***P*** for trend
**Multivariate COX Regression**				
**Cases**	**183**	**89**	**85**	
**Person-years**	**573,524**	**264,335**	**197,180**	
**Model 1**	**1.00(Ref.)**	**1.08 (0.84 ~ 1.40)**	**1.38 (1.06 ~ 1.81)**	**0.0616**
**Model 2**	**1.00(Ref.)**	**1.09 (0.84 ~ 1.41)**	**1.41 (1.08 ~ 1.85)**	**0.0428**
**Model 3**	**1.00(Ref.)**	**1.07 (0.82 ~ 1.38)**	**1.51 (1.15 ~ 1.98)**	**0.0121**
**CS Model**				
**Cases**	**183**	**89**	**85**	
**Person-years**	**573,524**	**264,335**	**197,180**	
**Model 1**	**1.00(Ref.)**	**1.08 (0.84 ~ 1.40)**	**1.38 (1.06 ~ 1.81)**	**0.0616**
**Model 2**	**1.00(Ref.)**	**1.08 (0.84 ~ 1.41)**	**1.41 (1.08 ~ 1.85)**	**0.0428**
**Model 3**	**1.00(Ref.)**	**1.06 (0.81 ~ 1.40)**	**1.50 (1.14 ~ 1.99)**	**0.0133**
**SD Model**				
**Cases**	**183**	**89**	**85**	
**Person-years**	**573,524**	**264,335**	**197,180**	
**Model 1**	**1.00(Ref.)**	**1.07 (0.83 ~ 1.39)**	**1.36 (1.04 ~ 1.79)**	**0.0780**
**Model 2**	**1.00(Ref.)**	**1.08 (0.84 ~ 1.40)**	**1.39 (1.06 ~ 1.82)**	**0.0554**
**Model 3**	**1.00(Ref.)**	**1.05 (0.80 ~ 1.40)**	**1.49 (1.13 ~ 1.98)**	**0.0151**

Model 1: Univariate analysis

Model 2: Adjusted for age, sex based on model 1

Model 3: Further adjusted for BMI, ALT, cirrhosis, hepatitis B virus infection, NAFLD, adiabetes mellitus, family history of cancer, smoking status, drinking status, physical activity based on model 2

CS model: In cause-specific hazard model; SD: sub-distribution hazard function model

### Association between hs-CRP and PLC risk in competing risk regression model

The results of the competing risk regression models were displayed in Table 2. There was an identification of 9232 death cases before the occurrent of PLC at the end of the follow-up period. Compared with the hs-CRP<1 mg/L group, the multivariable HRs (95%CI) for the association of hs-CRP of 1–3 mg/L group and hs-CRP>3 mg/L with PLC were 1.06(0.81 ~ 1.40), 1.50(1.14 ~ 1.99) in the multivariate-adjusted analysis in the CS model. Similar results were also observed in the SD model with minor differences.

### Subgroup analyses of association of hs-CRP with the risk of PLC

The effects of hs-CRP on new-onset PLC cases after stratifying the participants by sex, age, HBV infection, and cirrhosis were shown in Table 3. The effect of hs-CRP was not modified by sex both in the univariate and multivariate analyses. Similar results were also observed when participants were stratified by HBV infection or cirrhosis. However, when stratified by age, HR values increased only in the middle-aged and elderly groups.

**Table 3:** Hazard ratios and 95% confidence interval (CI) for risk of PLC among participants stratified by hs-CRP subgroups in different regression models

	hs-CRP				
	< 1 mg/L	1–3 mg/L	> 3 mg/L	***P*** for trend	***P*** for interaction
**Sex**					
**Men**	**1.00(Ref.)**	**1.14 (0.86 ~ 1.51)**	**1.52 (1.15 ~ 2.00)**	**0.0076**	**0.1433**
**Women**	**1.00(Ref.)**	**1.03 (0.82 ~ 1.36)**	**1.13 (1.01 ~ 1.29)**	**0.0121**	
**Age**					
**≤ 45**	**1.00(Ref.)**	**1.25 (0.53 ~ 2.95)**	**1.38 (0.63 ~ 3.03)**	**0.7209**	**< 0.001**
**45 ~ 60**	**1.00(Ref.)**	**1.20 (0.84 ~ 1.70)**	**1.41 (1.01 ~ 2.00)**	**0.0199**	
**≥ 60**	**1.00(Ref.)**	**0.83 (0.48 ~ 1.44)**	**1.54 (1.02 ~ 2.11)**	**0.2235**	
**HBV infection**					
**(−)**	**1.00(Ref.)**	**0.94 (0.65 ~ 1.36)**	**1.46 (1.04 ~ 2.04)**	**0.0184**	**0.1570**
**(+)**	**1.00(Ref.)**	**1.57 (1.03 ~ 2.40)**	**1.76 (1.14 ~ 2.72)**	**0.0309**	
**Cirrhosis**					
**(−)**	**1.00(Ref.)**	**1.12 (0.85 ~ 1.49)**	**1.55 (1.18 ~ 2.03)**	**0.0037**	**0.1638**
**(+)**	**1.00(Ref.)**	**0.89 (0.60 ~ 1.31)**	**1.10 (1.01 ~ 1.21)**	**0.0079**	

All analyses were adjusted for age, BMI, ALT, NAFLD, HBV infection, cirrhosis, diabetes mellitus, family history of cancer, smoking status, drinking status and physical activity when participants were stratified by sex

All analyses were adjusted for sex, BMI, ALT, NAFLD, HBV infection, cirrhosis, diabetes mellitus, family history of cancer, smoking status, drinking status and physical activity when participants were stratified by age

All analyses were adjusted for sex, age, BMI, ALT, NAFLD, cirrhosis, diabetes mellitus, family history of cancer, smoking status, drinking status and physical activity when participants were stratified by HBV infection

All analyses were adjusted for sex, age, BMI, ALT, NAFLD, HBV infection, diabetes mellitus, family history of cancer, smoking status, drinking status and physical activity when participants were stratified by cirrhosis

## Discussion

This prospective cohort study explored the relationship between hs-CRP and new-onset PLC among 95,759 Chinese participants. Overall, a positive correlation between hs-CRP levels and new-onset PLC was observed. Participants with hs-CRP > 3 mg/L were associated with a 51% increased risk of PLC compared with participants who had hs-CRP < 1 mg/L after adjustment was made for the traditional risk factors. However, such a relationship was not observed among participants with hs-CRP levels fell between 1 and 3 mg/L. This suggests that hs-CRP levels have to reach a threshold to be a potential risk factor for PLC. The risk of PLC also increased with the increase of hs-CRP when participants were stratified by sex, HBV infection, and cirrhosis. However, our study failed to find a positive relationship between hs-CRP and the risk of PLC among young participants. PLC is more common in elder patients, and long-term exposure to risk factors could explain the positive relation between hs-CRP and the risk of PLC in the middle-aged and old participants. The results are similar to the observations made in a nested case-control study in 2014 using data from the European Prospective Investigation into Cancer and Nutrition (EPIC) comprising of 10 European countries [16]. The study found that higher concentrations of CRP were positively associated with an elevated risk of hepatocellular cancer. After adjusting for confounding factors (inclusive of lifestyle factors, diabetes, hepatitis infection, and adiposity measures), the incidence rate ratio per doubling of concentration for CRP was 1.22, with 95% CI of 1.02–1.46. Similarly, another nested case-control study in 2015 found a significant association between higher serum CRP and elevated risk of liver cancer incidence as well as chronic liver disease mortality [18]. A statistically significant monotonic trend (*P* = 0.01) was found and subjects in the fourth quartile of CRP levels had a 63% higher risk than those in the first quartile. However, the association between CRP and liver cancer incidence was only seen in the males.

The increment of CRP has also been associated with more advanced and severe PLC. Hashimoto et al. first found that the preoperative CRP level was an independent and significant indicator that could accurately predict poor prognosis and early recurrence in PLC patients after hepatic resection [22]. A subsequent retrospective study on HCC patients with different stages by Nagaoka et al. concluded that overall, HCC patients with elevated CRP had a poorer prognosis than those with normal CRP levels [23]. Another prospective analysis for a cohort of 133 patients who were newly diagnosed with HCC similarly found that overall survival rates in the high CRP group were significantly lower than their counterparts in the low CRP group [24]. A study published in 2007 investigated the molecular mechanism of CRP in HCC cells in vitro, and concluded that CRP is highly expressed in tumor tissues, and also promotes invasion and metastases in HCC cell lines [25]. It has since been suggested that the addition of CRP to validated PLC staging systems could improve prognostic ability [26].

Hs-CRP has been established as an independent cardiovascular risk factor. However, the pathophysiological mechanism behind diseases with and without myocardial tissue damage differs. In the former situation, myocardial necrosis causes an acute phase response to be activated thereby increasing CRP, whereas CRP levels in cardiac diseases without myocardial tissue are dependent on the severity of atherosclerosis and other cardiovascular risk factors [15]. Yet even after adjustments were made for risk factors associated with cardiovascular diseases, a study in 1997 found that higher levels of CRP were still statistically significant when predicting increased risk of future myocardial infarction and stroke in apparently healthy participants [27], and the researchers further suggested that a chronic process is involved in mediating the effects of inflammation [14].

This idea of hs-CRP as a long-term marker of risk has also been applied to cancer. Allin et al. observed 10,408 individuals over a median follow-up period of 16 years and found that elevated baseline levels of CRP in cancer-free participants were associated with increased risk of lung and possibly colorectal cancer [28]. A later study by Aillin et al. further concluded that when maximizing sensitivity and specificity (both 61%) for prediction of lung cancer, optimal cut-point for CRP as a risk factor was 2.1 mg/L [29]. Although Aillin’s team did not find a statistically significant association between higher CRP levels and risk of colorectal cancer, a positive correlation between CRP levels and colorectal cancer metastases was found [28]. Controversy regarding an association between CRP and other types of cancer, namely prostate and breast cancer, also exists [30, 31]. The discrepancy arises because CRP is a non-specific marker and the biological mechanism of carcinogenesis in different organs varies. Additionally, many cancer cells produce CRP and it can also be difficult to pinpoint whether increase of CRP happens prior to the occurrence of cancer cells [32].

In the current study, the SD model and CS model applied to our analysis demonstrated a 1.5-fold and 1.49-fold increase in the risk of PLC for participants with hs-CRP > 3 mg/L compared with those with hs-CRP<1 mg/L. Despite their substantial differences, both models confirmed a positive relationship between higher levels of hs-CRP and new-onset PLC. Interestingly, there was not a significant difference between the COX regression model and the competing risk models, suggesting that death (competing event) have little impact on the estimation of risk of PLC associated with CRP level, and traditional COX regression analyses could estimate the actual individual risk properly.

CRP, an acute phase reactant, is commonly detected in inflammatory, infectious and tissue damage circumstances as a non-specific acute phase protein. For routine monitoring of infectious status, CRP concentration is usually measured using immunoturbidimetric and nephelometric methods, which have the detection limits of 3–5 mg/L. The development of ultrasensitive ELISA or particle-enhanced techniques has allowed the detection limits of CRP concentration (hs-CRP) to be less than 0.3 mg/L [33, 34] Unlike CRP, hs-CRP is typically recognized as an indicator of upcoming stroke, peripheral vascular disease and acute myocardial infarction. Though CRP and hs-CRP are the same entity, they have different analytical sensitivities, assay ranges and clinical significance.

The pathophysiological mechanism of the association between elevated hs-CRP concentrations and the risk of PLC is not fully elucidated. CRP is an acute-phase reactant and is a marker for systemic inflammation. It’s role in detecting or predicting inflammation outcomes is commonly applied clinically. Synthesis of CRP is mainly carried out in the liver as an inflammatory response to IL-6 secretion [35], and its pathophysiologic role during inflammation comprises of the ability to recognize some foreign pathogens, opsonize and active the complement system, initiate the elimination of targeted cells and also stimulate tissue factor in monocytes [16, 17]. It has recently been suggested that chronic inflammation is linked to PLC via multiple signaling pathways, some of which include NF-êB, c-jun, and STAT3. Complex interaction can sometimes occur within these pathways when liver damage-mediated inflammation and carcinogenesis are present [36].

HBV accounts for 7.2% of the Chinese general population and is the main etiology for PLC. In 2007, Hao et al. found that HBV infection upregulated the expression of the CRP gene both in vivo and in vitro [37]. In 2018, Shin et al. indicated that CRP immunoreactivity in non-neoplastic hepatocytes could be used as a prognostic biomarker of HBV-associated HCC, and indicated its association with certain tumor growth characteristics [38]. Although preliminary, these studies suggest the correlation between CRP and HBV. In this study, HBV was adjusted in multivariate analysis. Nonetheless, this did not attenuate the positive trend of hs-CRP and new on-set PLC, suggesting that CRP levels itself may be related to PLC incidence, regardless of its effect on HBV.

Strengths of our study include the prospective design, the sizable participant sample size (involving 95,759 participants), and a long median follow-up period (11.07 years per participant). Furthermore, baseline hs-CRP information and other PLC risk factor information also allowed us to make adjustments for suspected aforementioned confounders and analyze the long-term absolute effects of CRP on new-onset PLC. Additional strengths of the current study included almost 100% of the follow-up rate and the use of competing risk models, which may be more appropriate when assessing the risk of PLC, especially in the presence of multiple competing events.

Several limitations should be noted when interpreting the results of this study. First, exposure hs-CRP levels as well as other baseline assessments were only measured once and could have been influenced due to ongoing infection or medication use, allowing for potential misclassification which could skew our data analysis, especially in the long period of follow up. However, previous evidence has demonstrated CRP levels are stable over long periods with little variation [39]. Secondly, no data were available regarding HCV infection, which could have impacted the results. We hypothesize that this impact would not have been significant as HCV has a much smaller effect on the development of PLC in Chinese as compared to other Asian populations [40]. Third, because of the industrial characteristic of Kailuan Group, significantly more men than women were enrolled in this study. However, the influence of sex imbalance was minimized as analyses were further stratified by sex.

## Conclusion

Our results further provide evidence that chronic inflammation leading to higher levels of hs-CRP plays an important role in PLC and supports the notion that hs-CRP can be used as a risk factor for PLC. The main clinical implications would be an increased awareness of hs-CRP and its correlation to the risk of PLC. This study should be a steppingstone to further research on chronic inflammation and PLC.

## Data Availability

The data that support the findings of this study are available from Aerospace Center Hospital, but restrictions apply to the availability of these data, which were used under license for the current study, and so are not publicly available. Data are however available from the authors upon reasonable request and with permission of Aerospace Center Hospital.

## Abbreviations

PLC: Primary liver cancer
HCC: Hepatocellular carcinoma
ICC: Intrahepatic cholangiocarcinoma
HBV: Hepatitis B virus
HCV: Hepatitis C virus
hs-CRP: High-sensitivity C-reactive protein
WC: Waist circumference
BMI: Body mass index
TC: Total cholesterol
TG: Total glycerides
FGB: Fasting blood glucose
ALT: Alanine aminotransferase
HDL: High-density lipids
LDL: Low-density lipids
SBP: Systolic blood pressure
DBP: Diastolic blood pressure
NAFLD: Nonalcoholic fatty liver disease
ANOVA: One-way analysis of variance
HRs: Hazard ratios
CS model: Cause-specific hazard models
SD model: Sub-distribution hazard function model
RCS: Restricted cubic spline regression
